# Learning Curve of Robotic Pancreaticoduodenectomy with Portal–Superior Mesenteric Vein Resection for Pancreatic Cancers

**DOI:** 10.3390/jcm14227986

**Published:** 2025-11-11

**Authors:** Peng-Yu Ku, Yi-Ju Chen, Hui-Chen Lin, Yung-Hsien Chen, Sheng-Yang Huang

**Affiliations:** 1 Division of General Surgery, Department of Surgery, Taichung Veterans General Hospital, Taichung 407, Taiwan; lukephil6@gmail.com (P.-Y.K.); joaquinmaxo@yahoo.com.tw (H.-C.L.); Newmotlesimilu@gmail.com (Y.-H.C.); 2 Department of Post-Baccalaureate Medicine, College of Medicine, National Chung Hsing University, Taichung 402, Taiwan; 3 Division of Pediatric Surgery, Department of Surgery, Taichung Veterans General Hospital, Taichung 402, Taiwan; 4 School of Medicine, National Yang Ming Chiao Tung University, Taipei 112, Taiwan

**Keywords:** learning curve, pancreas cancer, pancreatoduodenectomy, robotic pancreatoduodenectomy, vein resection

## Abstract

**Background**: Pancreaticoduodenectomy (PD) with portal–superior mesenteric vein (PV-SMV) resection is increasingly performed in borderline-resectable periampullary cancer. While conventional PD is the reference standard, robotic PD (RPD) may improve operative ergonomics and recovery; its performance and learning curve in PV-SMV resection remain unclear. **Materials and Methods**: We retrospectively reviewed consecutive patients undergoing PD with PV-SMV resection at a single tertiary center by a single surgeon (July 2016–September 2022). Twenty-seven patients met the inclusion criteria and were grouped as conventional PD (*n* = 14) or RPD (*n* = 13). To assess the learning curve, RPD cases were stratified as early (cases 1–3) versus late (cases 4–13). Primary outcomes were operative time and blood loss; secondary outcomes included 90-day morbidity/mortality, R0 margin, lymph node yield, length of stay, readmission, and overall survival. **Results**: Baseline characteristics were comparable between conventional PD and RPD. Median operative time was longer with RPD vs. conventional PD (624.0 [IQR 579.0–794.0] vs. 529.5 [456.5–636.5] mins; *p* = 0.024). Median blood loss trended lower with RPD (350.0 [200.0–1950.0] vs. 1455.0 [630.0–2940.0] mL; *p* = 0.254). Rates of clinically relevant complications (including POPF, DGE, and hemorrhage), R0 resection (69% vs. 64%), lymph node retrieval, length of stay, 90-day readmission, 90-day mortality, and overall survival were similar between conventional PD and RPD. Within RPD, operative time and blood loss improved from the early to late phases (794.0 → 601.5 min; 1950.0 → 275.0 mL), consistent with a learning-curve effect, though not statistically significant in this small cohort. **Conclusions**: In selected patients, RPD with PV-SMV resection is feasible and achieves oncologic and short-term clinical outcomes comparable to conventional PD, with evidence of efficiency gains as experience accrues. These findings support structured training and case accumulation for the safe adoption of complex robotic pancreatic surgery.

## 1. Introduction

Pancreatic ductal adenocarcinoma (PDAC) remains a lethal malignancy with rising mortality worldwide and in the United States, despite incremental gains against other cancers [1]. Only a minority of patients are operable at presentation and five-year overall survival for unresectable disease remains <5% [1]. Even among candidates for curative surgery, outcomes are constrained by tumor proximity to major vessels and by perioperative risk [2,3,4,5].

Pancreaticoduodenectomy (PD) is the primary curative treatment for periampullary cancer. Operability depends on vascular involvement of the superior mesenteric artery, celiac axis, and the portal–superior mesenteric vein (PV-SMV) complex, which stratifies patients as borderline resectable or locally unresectable [4]. PD with PV-SMV resection has moved from controversy to conditional acceptance. Society endorsements and ISGPS guidance support its use when negative margins and safe venous reconstruction are achievable. Nevertheless, its comparative effectiveness and risk profile versus standard PD remain debated [6,7,8,9,10,11].

Minimally invasive pancreatoduodenectomy (MIPD), particularly robotic pancreatoduodenectomy (RPD), may improve dexterity, precision, and recovery while maintaining oncologic quality [12,13,14,15]. Nevertheless, adoption is tempered by technical complexity, the need for programmatic volume (≥20 MIPD/year), and a substantial learning curve for vascular resection (≈35 cases) [13,16]. Data specifically evaluating RPD with PV-SMV resection (RPD-VR) are limited, especially regarding perioperative safety, oncologic adequacy, and learning-curve dynamics [12].

This study is to characterize the learning curve using cumulative sum (CUSUM) and segmented regression methods and to compare perioperative and oncologic outcomes between RPD and open PD. We hypothesize that, in experienced programs, RPD-VR achieves perioperative and oncologic outcomes comparable to standard PD/MIPD while demonstrating measurable improvements across the learning curve.

## 2. Materials and Methods

**Figure 1:**
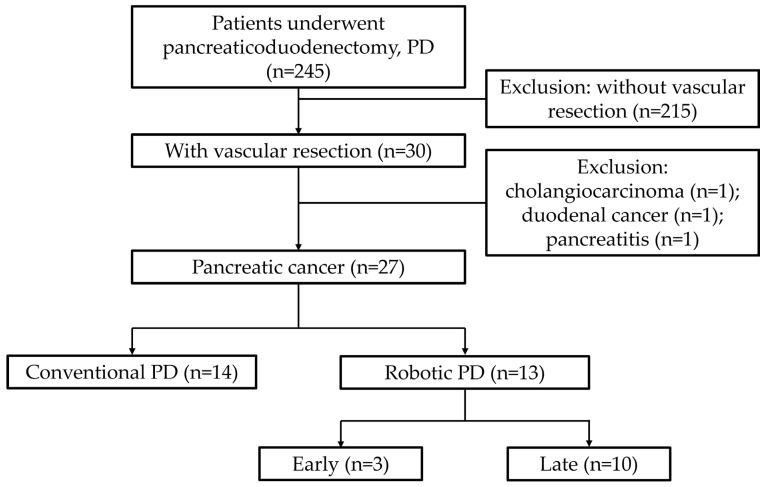
Flow diagram showing the selection of 245 patients undergoing pancreaticoduodenectomy (PD). Thirty underwent vascular resection. After exclusion of 1 cholangiocarcinoma, 1 duodenal cancer, and 1 pancreatitis case, patients were divided into conventional PD (*n* = 14) and robotic PD (*n* = 13). The Robotic group was further subdivided into early (*n* = 3) and late (*n* = 10).

The study retrospectively analyzed consecutive 245 patients who underwent PD at our institute. Among them, thirty patients had vascular resection during operation. Three cases were excluded based on etiology. The final analysis included 27 cases involving PV-SMV resection followed by reconstruction at the Division of General Surgery at Taichung Veterans General Hospital between July 2016 and September 2022. The pathological diagnosis was mainly pancreatic ductal adenocarcinoma, except for 1 intraductal papillary mucinous neoplasm with invasive carcinoma and 1 acinar cell carcinoma. Venous resection and reconstruction followed the four methods described by Kauffmann et al., including tangential resection with primary suture closure, artificial vessel patch reconstruction, segmental resection with primary end-to-end reconstruction, and artificial vessel interposition grafting [17]. All surgical patients were classified into two groups: 14 underwent conventional pancreaticoduodenectomy, while 13 underwent RPD conducted by a single experienced surgeon from July 2016 to September 2022 (Figure 1). For better comparison, patients were further divided into two groups: early stage cases (1–3) and late-stage cases (4–13). The study compared demographic data, intraoperative details, and postoperative outcomes between these groups. Perioperative drain management and diet advancement were not protocolized; rather, they were tailored to each patient based on the surgeon’s clinical judgment.

Clinical variables analyzed included patient sex, age, American Society of Anesthesiologists (ASA) classification, and body mass index (BMI) [18]. Surgical outcomes were assessed based on operative time, blood loss, conversion to open surgery, postoperative pancreatic fistula (POPF), bile leak, chyle leak, post-PD hemorrhage, delayed gastric emptying (DGE), abscess formation, wound infection, reoperation rate, Clavien–Dindo classification of complications, hospital stay duration, 90-day readmission rate, and 90-day operative mortality [19]. Pathologists documented tumor measurements, lymph node counts, and R0 resection status. According to ISGPS definitions for postoperative pancreatic fistula [20], delayed gastric emptying [21], and post-pancreatectomy hemorrhage [22], the complications were reported. Bile leak was defined as a drain-fluid bilirubin concentration ≥3 times the simultaneous serum level on or after postoperative day (POD) 3. Chyle leak was defined as milky fluid from the drain on or after POD3 with triglycerides ≥ 110 mg/dL.

Statistical analyses included the Mann–Whitney U test or Kruskal–Wallis test for non-normally distributed data, reported as median and interquartile range (IQR). Categorical data were analyzed using Chi-square and Fisher’s exact tests, with results expressed as frequencies and percentages. Survival analysis was conducted using the Kaplan–Meier estimator, with log-rank tests to compare survival curves between groups. The statistical computations were performed using IBM SPSS version 22.0 (International Business Machines Corp., New York, NY, USA). Data were presented as median (IQR) or number (percentage). For key contrasts (operative time, blood loss, and LOS), Hodges–Lehmann median differences and the effect sizes with 95%CIs were reported for key contrasts (operative time, blood loss, and LOS). No formal multiplicity control was performed across secondary endpoints or subgroup analyses; results are considered exploratory and hypothesis-generating. For learning curve evaluation, CUSUM charts were plotted in case order for two measures—operative time (mins) and intraoperative blood loss (mL). The CUSUM baseline was the cohort mean. Upward drift indicates values above the mean; downward drift indicates improvement versus the mean. Learning phases and change-points were determined not by CUSUM thresholds but by segmented linear regression; we compared models with segments and selected the best fit using the Bayesian Information Criterion (BIC). Each segment trend line was shown with 95% confidence intervals. Analyses were performed in Python 3.11.9 (Python Software Foundation, https://www.python.org/downloads/release/python-3119/, access on 11 August 2025) using matplotlib for figures.

## 3. Results

### 3.1. Conventional Versus Robotic

The conventional PD group comprised eight males and six females, while the RPD group included ten males and three females. The median age was 57.5 years (IQR: 55.2–67.8) in the conventional PD group and 62 years (IQR: 54.0–76.0) in the RPD group. ASA classification distributions were similar, with the conventional PD group having no ASA 1 cases, 11 ASA 2 cases, and 3 ASA 3 cases. The RPD group had one ASA 1 case, four ASA 2 cases, and eight ASA 3 cases. The median BMI was 21.8 kg/m^2^ (IQR: 19.6–23.2) in the conventional PD group and 22.8 kg/m^2^ (IQR: 21.4–24.1) in the RPD group. The groups were similar in demographic and clinical characteristics, except for operative time. The median operative time for the conventional PD group was 529.5 min (IQR: 456.5–636.5), whereas the RPD group required significantly more time at 624.0 min (IQR: 579.0–794.0). Robotic procedures resulted in less blood loss, but statistical tests indicated no significant difference compared to open procedures. The results are listed in Table 1.

For delayed gastric emptying, all patients had grade B conditions with the use of prokinetics. For patients with 90-day readmission, one patient with a distal metastasis lesion required biopsy, two were admitted for chemotherapy, one had post-chemotherapy neutropenia, one had ileus (conservative treatment), one had acute hepatitis of unknown etiology, and one had delayed gastric emptying with poor nutrition. One conversion case was noted in the robotic PD group due to massive bleeding (14,600 mL). The case underwent patch reconstruction for vein resection. Postoperative chemotherapy was applied for all patients in the conventional group (14/14, 100%) and most patients in robotic PD (10/13, 76.9%). One patient without adjuvant chemotherapy expired and the mortality rate was compatible with the patients with adjuvant chemotherapy (33.3% vs. 45.8%, *p* = 0.681).

Comparison between conventional PD and robotic PD stratified by ASA. The results are listed in Supplement Table S1. Baseline age/BMI and tumor size were similar across strata (age *p* = 0.268; BMI *p* = 0.780; tumor size *p* = 0.115). Operative time trended longer in Robotic ASA 3 cases (median 690.5 min), and LOS was shortest in robotic ASA 3 cases (median 18.0 days) (*p* = 0.069 and *p* = 0.447, respectively). Blood loss and lymph node yield did not differ (*p* = 0.531; *p* = 0.415). Major complications were rare: POPF, bile leak, and PPH were 0% in all groups; DGE ranged 12.5–40%. Conversion occurred in 1/5 (20%) Robotic ASA 1–2 cases. R0 rates were 54.5–100% with 0% 90-day mortality; readmission was highest in conventional ASA 1–2 cases (6/11, 54.5%).

**Figure 2:**
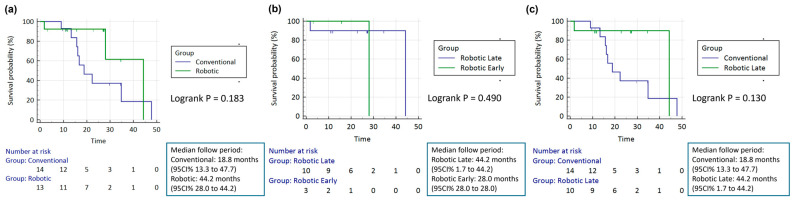
Kaplan–Meier overall survival of (**a**) conventional pancreaticoduodenectomy (PD) versus robotic; (**b**) robotic early versus robotic late; and (**c**) conventional PD versus robotic late. Overall survival probabilities are shown with log-rank test *p* values. Survival times are displayed in months, with curves starting at probability 100%.

Postoperative morbidity and mortality rates, including pancreatic fistula, bile leak, chyle leak, post-PD hemorrhage, delayed gastric emptying, abscess formation, wound infection, reoperation rate, Clavien–Dindo complications, hospital stay duration, 90-day readmission rate, and 90-day operative mortality showed no significant differences between groups. R0 resection rate and neoadjuvant therapy were about the same in both groups. Compared with the conventional PD (*n* = 14; nine mortality, five censored), robotic PD (*n* = 13; 3 events, 10 censored) showed no significant difference in overall survival (log-rank *p* = 0.183). Median follow-up was 18.8 months (conventional, 95%CI 13.3–47.7) and 44.2 months (robotic, 95%CI 28.0–44.2) (Figure 2a).

### 3.2. Early Stage Robotic Versus Late-Stage Robot

Further analysis divided the RPD group into early stage (cases 1–3) and late-stage (cases 4–13) subgroups. No significant demographic differences were observed between these subgroups. The late-stage group exhibited shorter operative times and reduced blood loss, though statistical significance was not established. Postoperative morbidity, mortality, and pathological outcomes remained consistent across early and late-stage RPD cases (Table 2, Figure 2b). In the comparison between the robotic early (*n* = 3; one event, two censored) and robotic late (*n* = 10; two events, eight censored), survival did not differ significantly (log-rank *p* = 0.490). Median follow-up was 28.0 months (early, 95%CI 28.0–28.0) and 44.2 months (late, 95%CI 1.7–44.2).

### 3.3. Conventional Versus Late-Stage Robot

A comparative analysis between late-stage robotic and conventional PD patients revealed similar baseline characteristics (Table 3). The conventional PD group required a median operative time of 529.5 min (IQR: 456.5–636.5), compared to 601.5 min (IQR: 560.2–646.5) in the late-stage robotic group. The late-stage robotic group experienced less blood loss (275.0 mL, IQR: 87.5–1362.5) compared to the conventional PD group (1455.0 mL, IQR: 630.0–2940.0). While this trend suggests robotic surgery may lead to reduced blood loss, statistical significance was not reached. The positive surgical outcomes remained consistent between open surgery and late-stage robotic procedures. Tumor dimensions and lymph node retrieval counts were equivalent between groups. The conventional PD group (*n* = 14; nine events, five censored) and robotic late group (*n* = 10; two events, eight censored) had no significant difference in overall survival (log-rank *p* = 0.130). Median follow-up was 18.8 months (95%CI 13.3–47.7) and 44.2 months (95%CI 1.7–44.2), respectively (Figure 2c).

### 3.4. Learning Curve for Robotic PD

**Figure 3:**
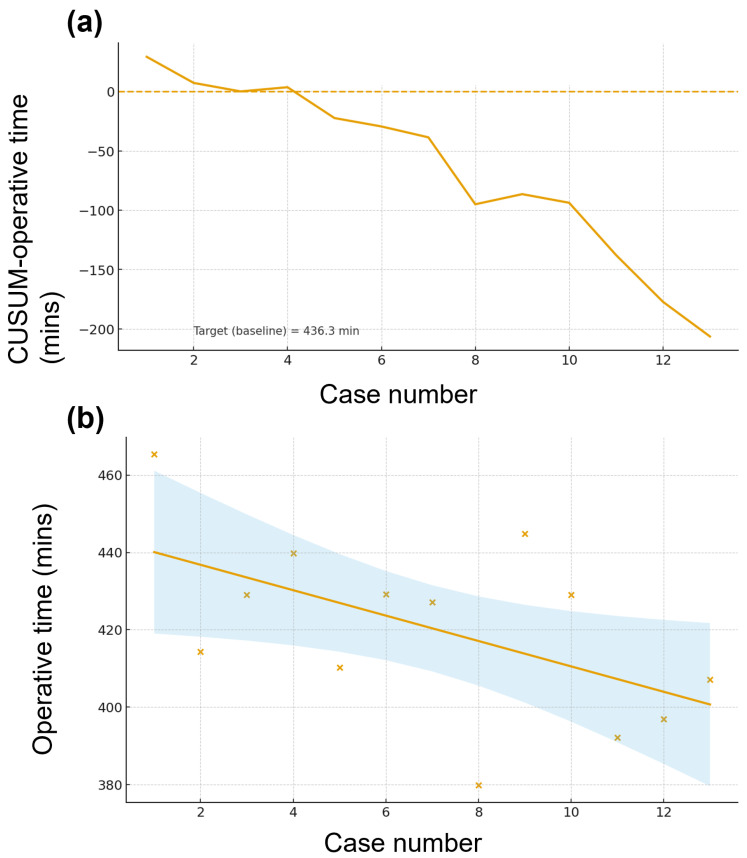
Learning curve of robotic pancreaticoduodenectomy (PD) cases with CUSUM and segmented learning-curve analysis. (**a**) Operative time—a CUSUM plot illustrating cumulative deviations from the baseline target; (**b**) operative time—a segmented learning-curve plot showing per-case observations, segment-wise fitted trends, 95%CIs, and estimated phase transitions (vertical dashed lines).

CUSUM curves for both operative time (Figure 3) and blood loss (Supplement Figure S1) trended downward, indicating performance better than the cohort mean targets (operative time 436.3 min; blood loss 511 mL). Linear modeling showed a progressive decrease in operative time across the first 13 cases, while segmented regression for blood loss identified a single changepoint at case 6 followed by a steeper decline.

### 3.5. Comparison Between Different Vein Reconstructions

A comparative analysis between different vein resection and repair methods is listed (Table 4). Across reconstruction strategies, only BMI and operative time differed significantly. Patients undergoing simple repair had the lowest BMI (median 19.5 kg/m^2^ vs. 23.3 interposition and 22.8 patch; *p* = 0.023). Operative time was longest with patch repair (639.0 min [572.8–743.8]) compared with simple repair (499.5 min [427.5–531.8]) and interposition graft (494.0 min [444.0–668.0]; *p* = 0.011). Blood loss and length of stay did not differ statistically, although medians were numerically higher for interposition and patch (blood loss 1700 and 1550 mL vs. 840 mL for simple repair; *p* = 0.472). Lymph node retrieval showed a non-significant trend toward fewer nodes with interposition grafts (median 8 vs. 15 simple and 13.5 patch; *p* = 0.083) and ASA class skewed toward higher risk in interposition/patch groups (1–2 vs. 3; *p* = 0.065). Rates of conversion (rare; 1/16 in patch), postoperative complications, DGE, infections, re-operation, 90-day readmission, and 90-day mortality were comparable across groups. R0 resection rates were similar (83.3% simple, 60.0% interposition, 62.5% patch; *p* = 0.614).

## 4. Discussion

Robotic assistance reproduces open techniques in a minimally invasive setting and enables an artery-first strategy that clarifies the posteromedial margin while supporting no-touch resection through selective ligation, suturing, and clipping of pancreaticoduodenal branches [12,23,24]. After adopting artery-first PD in 2019, our RPD outcomes improved, although late-phase RPD still required longer operative times than conventional PD, consistent with Beane et al. and Kauffmann et al. [6,13,17]. CUSUM analysis demonstrated progressive performance gains after the initial cases. For operative time, the CUSUM curve turned downward after case ~6–7 and reached a cumulative deficit by case 13, indicating sustained efficiency gains relative to baseline. For blood loss, CUSUM likewise crossed below zero early and declined steadily by case 13, consistent with improved hemostasis over time. Simple regression showed a negative trend for operative time with a 95%CI envelope suggesting a progressive per-case reduction. Segmented regression for blood loss identified an inflection around case 6: the early phase displayed greater variability and higher losses, whereas the late phase (≥7) showed a markedly steeper negative slope with narrower 95%CIs, indicating more consistent and rapidly improving blood-loss control.

According to the published learning-curve data, we propose a staged training pathway with explicit thresholds that preserve the original citation sequence. First, anchor program expectations to the observation that outcomes generally stabilize after ~20–40 RPD cases [25,26]. Within the earliest phase—roughly the first 10 cases, a high-risk period [27] that overlaps a 3–5-case “adaptation” window [28]—trainees should complete ≥10 dual-console and proctored RPDs under strict selection (exclude arterial abutment; limit venous work to tangential or short <2 cm repairs). Next, programs should target continued accrual toward ~50 total major pancreatic resections, consistent with reported improvements in morbidity, fistula, blood loss, and operative time after surpassing ~50 OPD cases [29], and expect performance consistency to emerge by ~40–60 RPDs [30] before broadly expanding indications. For venous reconstruction, we recommend that, after 10–15 standard RPDs, trainees undertake ≥10 supervised RPD-VR cases and aim for ~35 cumulative RPD-VR cases before independent practice [13]. Though our CUSUM turning point at case ~6–7 occurs earlier than stabilization thresholds commonly reported, these findings, along with our experience, emphasize the importance of experience and specialized training in achieving optimal surgical outcomes.

Our venous resection–reconstruction data indicate that patch repair is associated with longer operative duration, likely reflecting greater technical complexity, whereas short-term safety outcomes and margin status were comparable across reconstruction strategies, allowing techniques to be tailored to the defect. Practical adoption should be staged: begin with conservative case selection (initially excluding borderline-resectable disease), standardize an artery-first, no-touch approach, predefine PV-SMV reconstruction algorithms, and enforce explicit conversion thresholds within a team-based training pathway that meets recommended annual MIPD volumes to shorten the learning curve [12,13,16,23,24]. We acknowledge that institutional experience and deliberate early exclusion of highly complex cases can make early outcomes appear more favorable—effectively shortening the “observed” learning curve—thereby introducing selection bias. With these elements in place, our data suggest that robotic procedures reduce intraoperative blood loss without compromising oncologic endpoints compared with open surgery. We recommend dual-console proctoring for the first 3–5 RPDs and limiting early cases to those without arterial abutment and with venous defects amenable to tangential resection or short (<2 cm) primary/patch repair.

Limitations include retrospective design, selection bias, single-center setting, and short follow-up. This single-surgeon series is susceptible to temporal and selection confounding as case mix and perioperative care evolved alongside experience, without risk adjustment. We did not adjust the learning curve for calendar time; thus, secular trends in case mix or technology could confound the observed learning pattern. Residual confounding by baseline anesthetic risk is possible. The ASA physical status may have differed between groups and our sample size limited robust adjustment; therefore, some of the observed differences could reflect underlying physiological reserve rather than the surgical approach itself. Multicenter, risk-adjusted analyses are needed to validate these findings. The analysis is hypothesis-generating and not powered to demonstrate equivalence. Nonetheless, this study—among the first from Taiwan focusing on RPD-VR—adds program-level evidence that experience, standardized techniques, and disciplined adoption are key to safe implementation.

## 5. Conclusions

The study shows that PD with PV-SMV resection achieves safe outcomes along with similar oncological results no matter which approach, between conventional and robotic techniques, is utilized for selected patients. RPD with PV-SMV resection appears technically feasible and may achieve comparable short-term outcomes to open PD in selected patients, though larger multicenter analyses are required to confirm learning-curve thresholds and oncologic safety.

## Figures and Tables

**Table 1:** Comparison between conventional pancreaticoduodenectomy (PD) and robotic PD.

Variable	Conventional PD (*n* = 14)	Robotic PD (*n* = 13)	Hodges–Lehmann Median Difference	*p* Value
Sex (male/female)	8/6	10/3		0.420
Age (years)	57.5 (55.2–67.8)	62.0 (54.0–76.0)	4.00 (95%CI: −7.00 to 14.00)	0.576
ASA 1/2/3	0/11/3	1/4/8		0.054 (dichotomized 1&2 vs. 3)
BMI (kg/m^2^)	21.8 (19.6–23.2)	22.8 (21.4–24.1)	0.94 (95%CI: −1.09 to 3.20)	0.356
OP time (mins)	529.5 (456.5–636.5)	624.0 (579.0–794.0)	123.0 (95%CI: 29.5 to 249.0)	0.024
Blood loss (mL)	1455.0 (630.0–2940.0)	350.0 (200.0–1950.0)	−530 (95%CI: −1940.0 to 740.0)	0.254
LOS (days)	23.0 (17.0–29.8)	19.0 (17.0–24.0)	−1 (95%CI: −8.0 to 3.0)	0.557
Tumor size (cm)	4.0 (3.0–4.5)	3.0 (3.0–3.3)	−0.80 (95%CI: −1.75 to 0.30)	0.230
LNs retrieved	10.0 (7.2–15.0)	14.0 (10.0–19.0)	4.00 (95%CI: −1.00 to 10.00)	0.151
Conversion	0 (0%)	1 (8%)		0.481
POPF	0 (0%)	0 (0%)		1.000
Bile leak	0 (0%)	0 (0%)		1.000
Chyle leak	2 (14%)	2 (15%)		1.000
Post pancreatectomy hemorrhage	0 (0%)	0 (0%)		1.000
Delayed gastric emptying	5 (36%)	3 (23%)		0.678
Abscess	1 (7%)	0 (0%)		1.000
Wound infection	0 (0%)	1 (8%)		0.481
Re-operation	1 (7%)	0 (0%)		1.000
Clavien–Dindo ≥ 3	1 (7%)	0 (0%)		1.000
90-day readmission	6 (43%)	1 (8%)		0.077
90-day op mortality	0 (0%)	0 (0%)		1.000
R0 resection	9 (64%)	9 (69%)		1.000
Neoadjuvant	3 (21%)	3 (23%)		1.000

Continuous variables are shown as median (IQR) with *p* values from Mann–Whitney U test. Binary variables shown as *n* (%) with *p* values from Fisher’s exact test. ASA analyzed as dichotomized (1–2 vs. 3). R0 resection was defined as no residual tumor and free margin (>1 mm). ASA—American Society of Anesthesiologists classification, BMI—body mass index, POPF—postoperative pancreatic fistula, LOS—length of stay, LN—lymph node.

**Table 2:** Comparison between early and late stages in robotic pancreaticoduodenectomy.

Variable	Robotic Early (*n* = 3)	Robotic Late (*n* = 10)	Hodges–Lehmann Median Difference	*p* Value
Sex (male/female)	2/1	8/2		1.000
Age (years)	76.0 (74.5–81.5)	61.0 (51.0–62.0)	−20.50 (95%CI: −27.51 to −10.00)	0.074
ASA (1&2 vs. 3)	0/3	5/5		0.231
BMI (kg/m^2^)	21.5 (21.5–22.3)	23.0 (20.3–24.2)	1.12 (95%CI: −1.88 to 2.74)	0.573
OP time (mins)	794.0 (760.5–799.0)	601.5 (560.2–646.5)	−171.5 (95%CI: −235.0 to −58.0)	0.150
Blood loss (mL)	1950.0 (1675.0–2375.0)	275.0 (87.5–1362.5)	−1350 (95%CI: −2550.0 to 750.0)	0.202
LOS (days)	19.0 (18.0–20.0)	19.0 (17.0–24.8)	0.5 (95%CI: −3.0 to 8.0)	0.864
Tumor size (cm)	3.0 (3.0–4.0)	3.0 (2.4–3.3)	−0.25 (95%CI: −2.00 to 0.25)	0.662
LNs retrieved	10.0 (9.0–18.0)	14.5 (12.2–18.0)	4.00 (95%CI: −12.00 to 11.00)	0.498
Conversion	0 (0%)	1 (10%)		1.000
POPF	0 (0%)	0 (0%)		1.000
Bile leak	0 (0%)	0 (0%)		1.000
Chyle leak	1 (33%)	1 (10%)		0.423
Post pancreatectomy hemorrhage	0 (0%)	0 (0%)		1.000
Delayed gastric emptying	1 (33%)	2 (20%)		1.000
Abscess	0 (0%)	0 (0%)		1.000
Wound infection	0 (0%)	1 (10%)		1.000
Re-operation	0 (0%)	0 (0%)		1.000
Clavien–Dindo ≥ 3	0 (0%)	0 (0%)		1.000
90-day readmission	0 (0%)	1 (10%)		1.000
90-day op mortality	0 (0%)	0 (0%)		1.000
R0 resection	3 (100%)	6 (60%)		0.497
Neoadjuvant	0 (0%)	3 (30%)		0.528

Continuous variables are shown as median (IQR) with *p* values from Mann–Whitney U test. Binary variables shown as *n* (%) with *p* values from Fisher’s exact test. ASA analyzed as dichotomized (1–2 vs. 3). R0 resection was defined as no residual tumor and free margin (>1 mm). ASA—American Society of Anesthesiologists classification, BMI—body mass index, POPF—postoperative pancreatic fistula, LOS—length of stay, LN—lymph node.

**Table 3:** Comparison between conventional pancreaticoduodenectomy (PD) and late-stage robotic PD.

Variable	Conventional PD (*n* = 14)	Robotic Late (*n* = 10)	Hodges–Lehmann Median Difference	*p* Value
Sex (male/female)	8/6	8/2		0.388
Age (years)	57.5 (55.2–67.8)	61.0 (51.0–62.0)	−3.50 (95%CI: −9.00 to 8.00)	0.703
ASA (1&2 vs. 3)	11/3	5/5		0.204
BMI (kg/m^2^)	21.8 (19.6–23.2)	23.0 (20.3–24.2)	1.18 (95%CI: −1.40 to 4.11)	0.319
OP time (mins)	529.5 (456.5–636.5)	601.5 (560.2–646.5)	87.5 (95%CI: −11.5 to 212.0)	0.095
Blood loss (mL)	1455.0 (630.0–2940.0)	275.0 (87.5–1362.5)	−625 (95%CI: −2300.0 to 490.0)	0.107
LOS (days)	23.0 (17.0–29.8)	19.0 (17.0–24.8)	−0.5 (95%CI: −8.0 to 4.0)	0.701
Tumor size (cm)	4.0 (3.0–4.5)	3.0 (2.4–3.3)	−0.80 (95%CI: −1.90 to 0.30)	0.185
LNs retrieved	10.0 (7.2–15.0)	14.5 (12.2–18.0)	4.00 (95%CI: −1.00 to 11.00)	0.127
Conversion	0 (0%)	1 (10%)		0.417
POPF	0 (0%)	0 (0%)		1.000
Bile leak	0 (0%)	0 (0%)		1.000
Chyle leak	2 (14%)	1 (10%)		1.000
Post pancreatectomy hemorrhage	0 (0%)	0 (0%)		1.000
Delayed gastric emptying	5 (36%)	2 (20%)		0.653
Abscess	1 (7%)	0 (0%)		1.000
Wound infection	0 (0%)	1 (10%)		0.417
Re-operation	1 (7%)	0 (0%)		1.000
Clavien–Dindo ≥ 3	1 (7%)	0 (0%)		1.000
90-day readmission	6 (43%)	1 (10%)		0.172
90-day op mortality	0 (0%)	0 (0%)		1.000
R0 resection	9 (64%)	6 (60%)		1.000
Neoadjuvant	3 (21%)	3 (30%)		0.665

Continuous variables are shown as median (IQR) with *p* values from Mann–Whitney U test. Binary variables shown as *n* (%) with *p* values from Fisher’s exact test. ASA analyzed as dichotomized (1–2 vs. 3). R0 was defined as no residual tumor and free margin (>1 mm). ASA—American Society of Anesthesiologists classification, BMI—body mass index, POPF—postoperative pancreatic fistula, LOS—length of stay, LN—lymph node.

**Table 4:** Comparison between vein reconstruction types.

Variable	Simple Repair (*n* = 6)	Interposition Graft (*n* = 5)	Patch (*n* = 16)	*p* Value
Sex (male/female)	3/3	3/2	12/4	0.509
Age (years)	60.0 (57.2–65.8)	57.0 (55.0–66.0)	62.0 (54.8–73.8)	0.642
ASA (1&2 vs. 3)	6/0	2/3	8/8	0.065
BMI (kg/m^2^)	19.5 (18.7–19.7)	23.3 (21.1–23.9)	22.8 (21.5–24.2)	0.023
OP time (mins)	499.5 (427.5–531.8)	494.0 (444.0–668.0)	639.0 (572.8–743.8)	0.011
Blood loss (mL)	840.0 (345.0–1132.5)	1700.0 (580.0–3890.0)	1550.0 (237.5–2770.0)	0.472
LOS (days)	20.5 (14.0–24.8)	24.0 (18.0–31.0)	19.5 (17.0–26.0)	0.470
Tumor size (cm)	3.0 (2.6–3.8)	4.5 (2.7–6.2)	3.2 (3.0–4.3)	0.762
LNs retrieved	15.0 (10.5–15.8)	8.0 (5.0–10.0)	13.5 (9.8–23.0)	0.083
Conversion	0 (0.0%)	0 (0.0%)	1 (6.2%)	0.991
POPF	0 (0.0%)	0 (0.0%)	0 (0.0%)	0.835
Bile leak	0 (0.0%)	0 (0.0%)	0 (0.0%)	0.835
Chyle leak	1 (16.7%)	1 (20.0%)	2 (12.5%)	0.909
Post pancreatectomy hemorrhage	0 (0.0%)	0 (0.0%)	0 (0.0%)	0.835
Delayed gastric emptying	1 (16.7%)	2 (40.0%)	5 (31.2%)	0.683
Abscess	0 (0.0%)	0 (0.0%)	1 (6.2%)	0.991
Wound infection	0 (0.0%)	0 (0.0%)	1 (6.2%)	0.991
Re-operation	0 (0.0%)	1 (20.0%)	0 (0.0%)	0.241
Clavien–Dindo ≥ 3	0 (0.0%)	0 (0.0%)	1 (6.2%)	0.991
90-day readmission	2 (33.3%)	2 (40.0%)	3 (18.8%)	0.572
90-day op mortality	0 (0.0%)	0 (0.0%)	0 (0%)	1.000
R0 resection	5 (83.3%)	3 (60.0%)	10 (62.5%)	0.614
Neoadjuvant	2 (33.3%)	1 (20.0%)	3 (18.8%)	0.758

Continuous variables are as median (IQR) with *p* values from Kruskal–Wallis test. Binary variables shown as *n* (%) with *p* values from Fisher’s exact test. ASA analyzed as dichotomized (1–2 vs. 3). R0 resection was defined as no residual tumor and free margin (>1 mm). ASA—American Society of Anesthesiologists classification, BMI—body mass index, POPF—postoperative pancreatic fistula, LOS—length of stay, LN—lymph node.

## Data Availability

The datasets generated and analyzed during the current study are not publicly available due to IRB restrictions but are available from the corresponding author on reasonable request. Access to the data will be provided upon personal contact with the corresponding author in accordance with applicable regulations and institutional policies.

## Supplementary Materials

The following supporting information can be downloaded at: https://www.mdpi.com/article/10.3390/jcm14227986/s1, Figure S1: Learning curve of robotic pancreaticoduodenectomy (PD) cases with CUSUM and segmented learning-curve analysis (a) Blood loss- a CUSUM plot illustrating cumulative deviations from the baseline target; (b) Blood loss- a segmented learning-curve plot showing per-case observations, segment-wise fitted trends, 95% CIs, and estimated phase transitions.; Table S1: Comparison between conventional pancreaticoduodenectomy (PD) and robotic PD stratified by ASA.

## Abbreviations

The following abbreviations are used in this manuscript:

PD	Pancreaticoduodenectomy
PV-SMV	Portal–superior mesenteric vein
RPD	Robotic pancreaticoduodenectomy
OS	Overall survival
MIPD	Minimally invasive pancreatoduodenectomy
ASA	American Society of Anesthesiologists
BMI	Body mass index
POPF	Postoperative pancreatic fistula
DGE	Delayed gastric emptying
IQR	Interquartile range
LOS	Length of stay
LN	Lymph node
RPD-VR	RPD with vascular resection
