# Supplementary material for: Learning Curve of Robotic Pancreaticoduodenectomy with Portal–Superior Mesenteric Vein Resection for Pancreatic Cancers
Source: J Clin Med. 2025 Nov 11;14(22):7986. doi: 10.3390/jcm14227986 (PMC12653503; doi:10.3390/jcm14227986)
Supplement: Supplementary file 1 [file jcm-14-07986-s001.zip › jcm-3905643-supplementary.pdf]

Supplement Table S1. Comparison between conventional pancreaticoduodenectomy (PD) and robotic PD stratified by ASA.

Variable	Conventional PD/ ASA 1 or 2 (n=11)	Conventional PD/ ASA 3 (n=3)	Robotic PD/ ASA 1 or 2 (n=5)	Robotic PD/ ASA 3 (n=8)	p value
Sex (male/female)	6/5	2/1	3/2	7/1	
Age (years)	58.0 (56.0-71.0)	55.0 (44.0-68.0)	54.0 (49.5-71.0)	67.5 (60.5-79.7)	0.268
BMI (kg/m^2)	22.1 (19.5-23.3)	21.0 (14.9-24.2)	23.1 (18.2-24.1)	22.3 (21.4-24.9)	0.780
OP time (mins)	534.0 (412.0-663.0)	494.0 (444.0-904.0)	554.0 (511.5-726.0)	690.5 (590.2-801.5)	0.069
Blood loss (mL)	1210.0 (580.0-3000.0)	1700.0 (530.0-6550.0)	250.0 (125.0-7450.0)	1550.0 (125.0-2587.5)	0.531
LOS (days)	22.0 (17.0-30.0)	24.0 (18.0-31.0)	24.0 (18.0-30.7)	18.0 (17.0-20.7)	0.447
Tumor size (cm)	4.1 (3.0-4.5)	2.7 (0.9-9.0)	3.0 (2.2-3.0)	3.2 (3.0-4.7)	0.115
LNs retrieved	10.0 (7.0-16.0)	10.0 (2.0-12.0)	13.0 (8.5-29.5)	14.5 (20.5-11.0)	0.415
Conversion	-	-	1 (20%)	0 (0%)	
POPF	0 (0%)	0 (0%)	0 (0%)	0 (0%)	
Bile leak	0 (0%)	0 (0%)	0 (0%)	0 (0%)	
Chyle leak	2 (18.1%)	0 (0%)	2 (40%)	0 (0%)	
Post pancreatectomy hemorrhage	0 (0%)	0 (0%)	0 (0%)	0 (0%)	
Delayed gastric emptying	4 (36.3%)	1 (33.3%)	2 (40.0%)	1 (12.5%)	
Abscess	1 (9%)	0 (0%)	0 (0%)	0 (0%)	
Wound infection	0 (0%)	0 (0%)	0 (0%)	1 (12.5%)	
Re-operation	1 (9%)	0 (0%)	0 (0%)	0 (0%)	
Clavien-Dindo ≥3	1 (9%)	0 (0%)	0 (0%)	0 (0%)	
90-day readmission	6 (54.5%)	0 (0%)	0 (0%)	1 (12.5%)	
90-day op mortality	0 (0%)	0 (0%)	0 (0%)	0 (0%)	
R0 resection	6 (54.5%)	3 (100.0%)	3 (60.0%)	6 (75.0%)	
Neoadjuvant	2 (18.1%)	1 (33.3%)	1 (20.0%)	2 (25.0%)	

[1] Continuous variables shown as median (IQR) with p values from Kruskal–Wallis test. Binary variables shown as n (%) with p values from Fisher's exact test. ASA analyzed as dichotomized (1–2 vs 3). R0 resection was defined as no residual tumor and free margin (>1mm).

[2] ASA American Society of Anesthesiologists classification, BMI body mass index, POPF postoperative pancreatic fistula, LOS length of stay, LN lymph node.

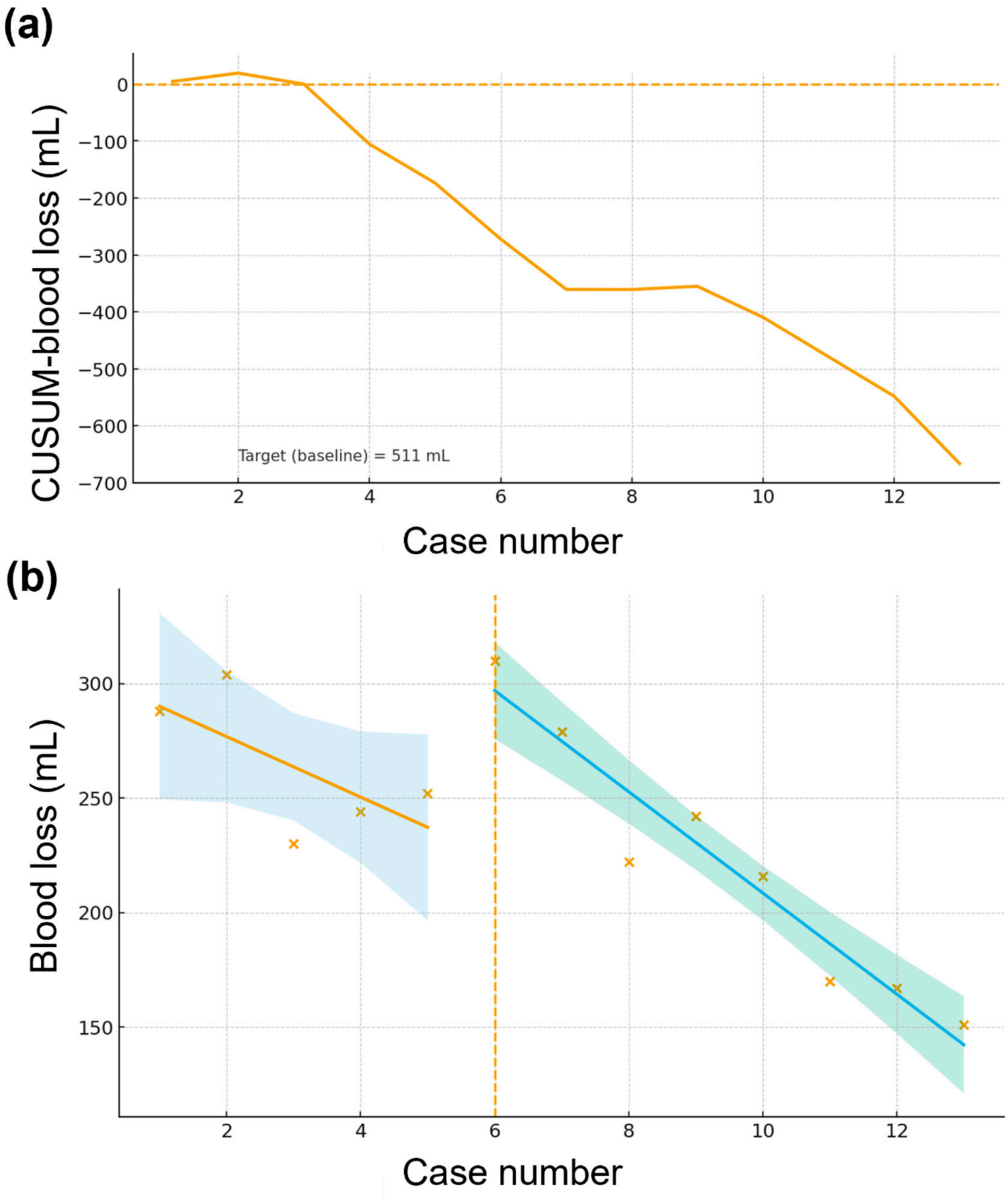

Supplement Figure S1. Learning curve of robotic pancreaticoduodenectomy (PD) cases with CUSUM and segmented learning-curve analysis (a) Blood loss- a CUSUM plot illustrating cumulative deviations from the baseline target; (b) Blood loss- a segmented learning-curve plot showing per-case observations, segment-wise fitted trends, 95% CIs, and estimated phase transitions.
